# Integrated Computational Material Design for PMC Manufacturing with Trapped Rubber

**DOI:** 10.3390/ma13173825

**Published:** 2020-08-29

**Authors:** Brina J. Blinzler, Pooria Khalili, Johan Ahlström

**Affiliations:** 1 Division of Material and Computational Mechanics, Department of Industrial and Materials Science, Chalmers University of Technology, SE-412 96 Gothenburg, Sweden; pooriak@chalmers.se; 2 Division of Engineering Materials, Department of Industrial and Materials Science, Chalmers University of Technology, SE-412 96 Gothenburg, Sweden; johan.ahlstrom@chalmers.se

**Keywords:** trapped rubber processing, composites, processing, simulation, elastomers

## Abstract

As the use of continuous fiber polymer matrix composites expands into new fields, there is a growing need for more sustainable manufacturing processes. An integrated computational material design framework has been developed, which enables the design of tailored manufacturing systems for polymer matrix composite materials as a sustainable alternative to achieving high-quality components in high-rate production. Trapped rubber processing achieves high pressures during polymer matrix composite processing, utilizing the thermally induced volume change of a nearly incompressible material inside a closed cavity mold. In this interdisciplinary study, the structural analysis, material science and manufacturing engineering perspectives are all combined to determine the mold mechanics, and the manufacturing process in a cohesive and iterative design loop. This study performs the coupled thermo-mechanical analysis required to simulate the transients involved in composite manufacturing and the results are compared with a previously developed test method. The internal surface pressure and temperatures are computed, compared with the experimental results, and the resulting design process is simulated. Overall, this approach maintains high-quality consolidation during curing while allowing for the possibility for custom distributions of pressures and temperatures. This can lead to more sustainable manufacturing by reducing energy consumption and improving throughput.

## 1. Introduction

**Figure 1:**
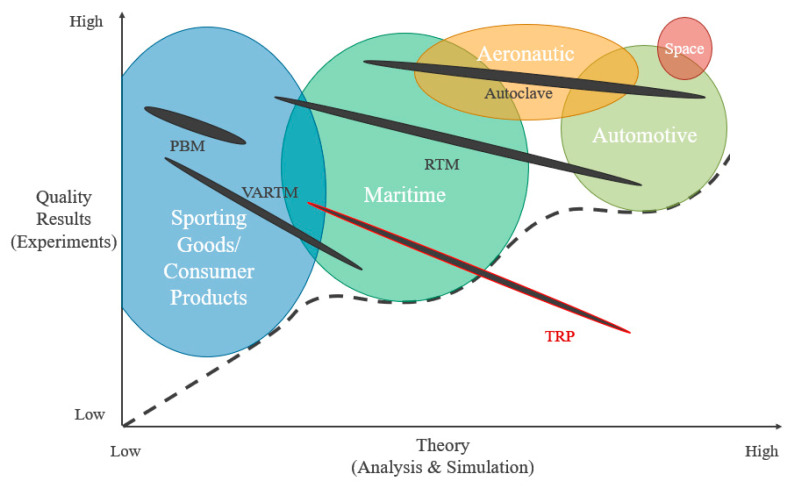
Boundary of trial and error manufacturing processes in polymer matrix composites (PMCs).

The need for autoclave alternatives for high-performance composite processing that allow faster throughput while maintaining performance becomes more pronounced as the demand for fiber-reinforced polymers (FRPs) increases. The current state of continuous fiber composite manufacturing for high-performance polymer matrix composites (PMCs) is dominated by manufacturing techniques that are straight forward to experimentally improve via an intensive trial and error process. In order to move beyond the regime of trial and error manufacturing processes (Figure 1), robust simulation methods must be established to model processing. In Figure 1, the theory is compared with the quality of experimental results for different processing methods, including the Trapped Rubber Processing (TRP) method studied in this paper. The term “Theory” on the *x*-axis represents the amount of effort expended to understand the structural analysis, material science and manufacturing engineering of PMCs. “Quality of results” indicated on the *y*-axis represents the complex experimental methods and structural design trees or number of full-scale tests needed to verify the robustness of a PMC component or structure design. High-performance PMCs (PMCs with fiber volume fractions greater than 50%) are generally used in five primary industries: sporting goods or consumer products, maritime structures, aeronautic structures, automotive components, and space structures [1,2,3]. In general, the easier it is to fabricate and test a design, the lower the need for advanced simulation. As the difficulty, size and cost of testing apparatus increases, more robust simulation procedures are required for the processing design. Each industry has developed a unique approach to structural design and processing design. A common aeronautic ‘building block’ approach to experimental testing and certification includes multiple levels of testing [3]. These range from the small-scale coupon testing to the large-scale component level [3]. The automotive industry includes full vehicle simulations in medium-to-large volume production runs, which leads to a greater emphasis on the computational characterization of the structural material response and the processing design characterization [1].

TRP can be used as a manufacturing method for both thermoplastic composite tape or comingled mat and thermoset pre-impregnated fiber preforms. Carbon or glass fiber combined with resins such as polyamide, polyether ether ketone, or autoclave grade epoxies are examples of these types of preform material options [4,5]. Processing temperatures vary with the resin material. Lower peak temperatures are required for epoxies (30–200 °C), while slightly higher temperatures are required for polyamides (190–300 °C) and even higher temperatures are required for polyether ether ketone (350–400 °C). Processing pressures vary and must be specifically chosen for the preform composite material. Maximum manufacturing pressures can range from atmospheric to 1800 kPa for these types of PMC preforms. Pressure schedule control is an important tool for maintaining quality in PMC components [1,2]. This is discussed in detail in the following paragraphs.

**Figure 2:**
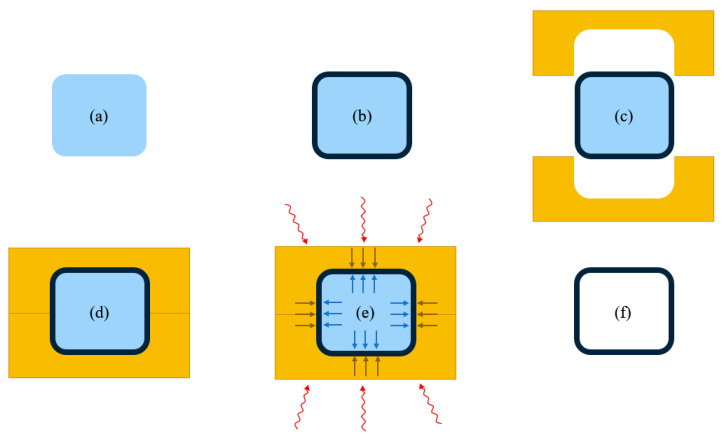
Schematic of trapped rubber processing (TRP) in a simplified cross-section view: (**a**) initial rubber section, (**b**) preform construction, (**c**) clamshell cavity mold, (**d**) processing mold assembly, (**e**) heating and pressure cycle, (**f**) demolding.

A simplified TRP workflow can be seen in Figure 2. Here, (a) shows the cross section of a three-dimensional rubber shape, the TRP unit. In the following step, (b), the PMC preform is draped, drawn or wrapped around the TRP unit. Next, a two-part exterior mold is fitted in place ((c) and (d)). The cure or melt cycle is executed in step (e). Finally, the PMC component is demolded (f). Trimming, painting or other processes can then follow. Figure 2 is only an example, another method used to manufacture kayak paddles applies the rubber to only one side of the preform surface [6].

In Figure 1, the boundary of trial and error PMC processing development can be visualized. In order to move beyond that boundary, an understanding of the fundamental constraints forming this boundary need to be gained. Two of the most common high-performance PMC manufacturing processes and their limitations are discussed in the following paragraphs, in order to further this understanding. One common method is autoclave processing of PMCs. This relies on polymer compaction through a combination of vacuum bagging and pressurization. The autoclave is heated in a way that increases temperature in the air and this air heats the surface of the part. Using this process, it can take hours for a PMC part to go through the full cure cycle [7,8]. Additionally, autoclaves must be sized for the largest part produced and are costly to both acquire and maintain [9]. Currently, there are a number of tools developed for the simulation of autoclave processing [10]. Pressurized bladder molding (PBM) is another processing method, where the composite preform is placed in a hard cavity mold and pressurized by inflating an internal bladder. This allows for the cure cycle to be reduced to under an hour for thin parts, but the process can be susceptible to bladder rupture and is typically used for small parts due to the cost of the exterior tooling [11,12]. Additionally, PBM uses a heating method that is applied at the exterior tooling, so there is limited possibility of internal heating to further reduce processing time. Vacuum-Assisted Resin Transfer Molding (VARTM) and Resin Transfer Molding (RTM) limit the volume fraction of the reinforcement, which can limit the effective performance of the material [1]. There has been a considerable amount of research conducted in the simulation of both VARTM and RTM processing [13,14].

Trapped rubber processing is not a manufacturing method that adapts well to the trial and error approach. In the mid-1970s, a number of polymeric elastomers were developed for TRP [15,16,17] but the research stalled due to the low thermal conductivity of the materials and the complex physics causing a disconnect between change in temperature and change in pressure during processing. Over-pressurization is a common initial problem when using a trial-and-error-based process design methodology for TRP. This can quickly result in broken molds and damaged equipment that hinders further processing improvements and ultimately the adoption of the manufacturing technique [6]. This makes it clear that an integrated computational material design framework is required for TRP processing design. Specifically, a well characterized rubber material model is needed that can be used in conjunction with existing process modeling methods [18]. Two recent advances in technology have increased the readiness of the TRP technique. Both advances in materials and computational capabilities further the possibility of manufacturing complex shapes and high-volume PMC production. Interest from the electronics industry has fueled extensive research in the development of a number of elastomers with high thermal conductivity. This increase in thermal conductivity is generally achieved by using nanoscale metallic additives [19] or conductive copolymers [20,21].

The elastomeric nanocomposites developed for the electronic and biomedical industries may have additional benefits for high-throughput PMC manufacturing. Non-thermoelastic residual stresses are developed during PMC manufacturing due to through-thickness degree of cure or crystallinity gradients [18]. These through-thickness cure gradients are exacerbated primarily by two mechanisms. One is the thickness of the composite preform and the other is the rate of thermal loading. Automated, high-throughput PMC manufacturing can require high-temperature curing, but sharp distortions are intensified by increasing the processing temperature range [22,23]. In-plane residual stresses are further accentuated by increasing the thickness of the composite preform [24,25,26,27]. For thick parts, it is more efficient (w.r.t. processing time) to processes the component in a single cycle. However, due to the effect of cure gradient on the residual stresses and other phenomena [27], typically, multiple cycles are used to processes parts greater than the resin manufacturer’s recommended cure thickness. Nano additives can be exploited to customize the thermal conductivity of the TRP material [18,21] and mitigate some thermal gradient effects. By tailoring the conductivity of the material, sections of the TRP unit in contact with thicker sections of the composite preform can be designed with a comparatively higher conductivity to those other sections in contact with thinner composite preform. Additive manufacturing is a technique used in biomedical research to functionally grade structures with tailored properties [28] and could potentially be used to manufactured TRP units. This has the potential to also eliminate locally under-cured or over-cured areas of the structure. There has also been investigation into copolymer formulations that combine the hyperplastic properties of one polymer with the high thermal conductivity properties of another polymer [20,22]. TRP materials, in general, are ideal candidates for nanoscale optimization of multifunctional mechanical and thermal properties.

Once the optimal thermal conductivity distribution is achieved, the remaining issue is how to link the temperature change with the dynamic change in pressure at the surface. Here, a solution is proposed. An integrated computational material design framework is proposed that uses a TRP characterization method to develop a material model for computational analysis. Using the results from the previously developed experimental characterization method [29] that captured the volumetric change in pressure via a series of tests, the material model can be calibrated for use in TRP modeling. The resulting surface pressure from the coupled thermomechanical simulation is then compared with the measured pressure on the exterior surface of the TRP Unit.

## 2. Materials and Methods

### 2.1. Modeling

The development of an intuitive and accurate simulation approach is an important part of the integrated computational material design framework presented in this paper. There are two main difficulties encountered when modeling TRP processing. One difficulty is the behavior of the hyperelastic material, particularly when confined, and the other is the coupled thermomechanical nature of the problem. Many elastomers have a very high bulk modulus in contrast to their relatively low shear modulus, which causes numerical sensitivities for three-dimensional solid elements. A number of models have been developed to analyze hyperelastic materials, such as silicone rubber. One of the empirically based approaches is the Mooney–Rivlin method [30,31]. This method is accurate up to 200% strain [32], which is outside the typical strain observed in TRP materials. Typical strains observed in TRP processing are greater than −7% in compression and, for the spherical assembly, are less than 1% in shear. For assemblies with large flat sections, the expected shear strain should be checked to verify that the assumptions still hold. The Mooney–Rivlin method can model the large strain and nonlinear behavior of incompressible materials such as rubber. This model consists of a series of parameters or curves calibrated to fit test data of a specific hyperelastic material. The Mooney–Rivlin model was selected as the initial material model for this study for its ease of implementation. It can be initialized in a relatively simply way by using the elastic modulus and the Poisson’s ratio as detailed in Section 2.2.

A fully coupled thermomechanical simulation is required for modeling TRP. The current thermal state contributes to the rubber volume, or rather in a confined case the pressure on the surface and the internal stress. The surface pressure simultaneously affects the thermal conductance at the interface. Due to this fully coupled nature, it is not surprising that the trial and error method of processing improvements presents such a challenge. Thermomechanical material modeling can be conducted in different ways. An extension to the Mooney–Rivlin method to incorporate the thermomechanical response of the hyperelastic material has been developed by Lion et al. [33]. In this method, they consider a hydrostatic compression case where a specific compression modulus parameter is defined for the material. While realistic TRP units will have a complex stress state, the simplified hollow sphere designed for use in the experiments has primarily hydrostatic stress. The design provides a straight-forward way to calibrate the initial material model. This method was selected for use in this study to make the simulation method straight forward to parameterize, robust, and simple to implement by utilizing a commercially available software (Abaqus/Standard v.2017 [34])and existing subroutines.

The finite element modeling in this study makes use of a fully coupled temperature-displacement analysis, which is performed in Abaqus/Standard [34]. The implementation of the thermomechanical analysis uses Newton’s method with a nonsymmetric Jacobian matrix, as shown in Equation (1). The variables Δu and Δϴ are the incremental displacement and temperature corrections, respectively, $K_{ij}$ are the submatrices of the fully coupled Jacobian matrix, and $R_{u}$ and $R_{ϴ}$ are the respective mechanical and thermal residual vectors [34].

(1) $$\left[\begin{array}{cc} K_{uu} & K_{uϴ}\\ K_{ϴu} & K_{ϴϴ} \end{array}\right]\left\{\begin{array}{c} \Delta u\\ \Delta ϴ \end{array}\right\}=\left\{\begin{array}{c} R_{u}\\ R_{ϴ} \end{array}\right\}$$

#### Molding Assembly

**Figure 3:**
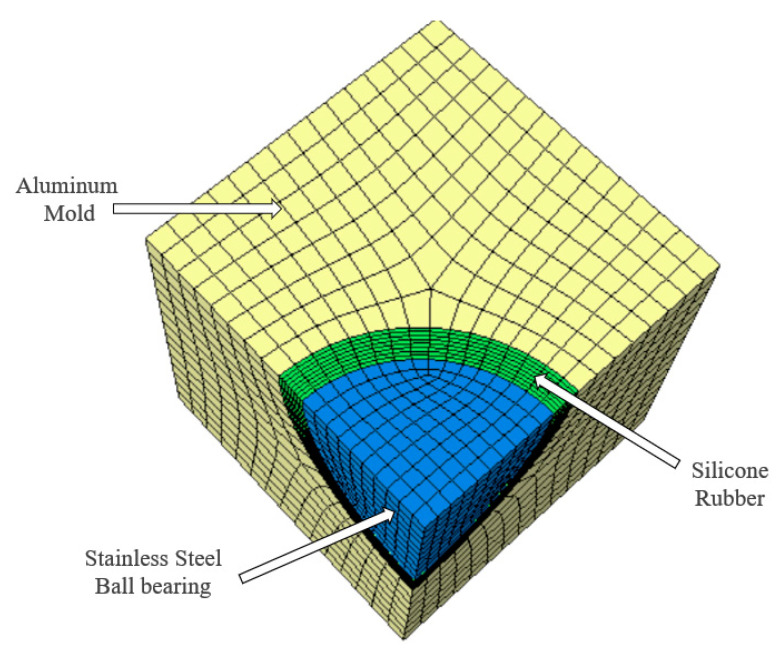
Molding assembly used in the simulation.

The simulation set-up is constructed to mimic the experimental set-up [29] and can be seen in Figure 3. A hollow rubber sphere is centered in a two-part cavity mold, where a stainless steel sphere constrains the inner diameter and the aluminum mold constrains the outer diameter. Heat is applied on the mold exterior to simulate the heating in the experiment [29]. Element and node sets are formulated to correlate with the location of sensors in the experimental set-up. In this way, the simulation can be compared directly to the experimental output.

The elements used for the stainless steel and aluminum sections in the molding assembly are C3D8RT (eight-node thermally–mechanically coupled hexahedral elements with reduced integration) [34]. The use of reduced integration elements reduces the total simulation runtime by approximately 30% and has a negligible effect on the results. The use of reduced integration elements resulted in a surface pressure that was less than 1% lower than the surface pressure obtained using fully integrated elements. The rubber material is modeled using Abaqus/Standard [34] C3D8HT (8-node thermally–mechanically coupled hexahedral, trilinear displacement and temperature, hourglass control, hybrid, constant pressure) elements, and the analysis is run in single precision. By using hybrid elements for the rubber, the runtime is decreased by over 30%, while resulting in an interface surface pressure that is approximately 1% lower than the simulation using the fully integrated elements with the hybrid formulation being marginally closer to the experimental results. There were no convergence issues with any of the formulations included in the study.

In nearly incompressible materials such as hyperelastics, small changes in displacement can produce extremely large changes in pressure. Due to this nature of the rubber materials used in TRP, standard element formulations produce an overly stiff model. It is well known that volumetric locking of incompressible materials in finite element analysis is most severe when the material is highly confined. However, the changes in volume can be calculated independently from the changes in shape as seen in Lion et al. [33]. The use of reduced integration can mitigate volumetric locking to some extent but combining this type of independent volume and shape calculation with reduced integration elements further prevents locking. The hybrid element formulation in Abaqus utilizes the separation of change in volume and change in shape, which results in the following expression for the rate of virtual work, Equation (2) [34]. It is recommended that materials with a Poisson’s Ratio of greater than 0.475 use the hybrid formulation [34]. As the Poisson’s ratio of the silicone rubber used in this study is 0.49, a combination of hybrid and reduced integration elements are used for the rubber material.

(2) $$\begin{array}{c} d\delta \bar{W}=\int_{V}\{\delta \varepsilon :C:\delta \varepsilon -\frac{1}{9K}\left(1-\rho \right)\delta \varepsilon :C^{T}:I\;I:C:d\varepsilon +\delta \varepsilon :\bar{\sigma }I:d\varepsilon \\ -\frac{1}{3K}\left(1-\rho \right)\left(\delta \hat{p}I:C:d\varepsilon +\delta \varepsilon :C^{T}:Id\hat{p}\right)-\frac{1}{K}\left(1-\rho \right)\delta \hat{p}d\hat{p}\\ +\bar{\sigma }:\left[{\left(\frac{\partial \delta u}{\partial x}\right)}^{T}\cdot \frac{\partial du}{\partial x}-2\delta \varepsilon \cdot d\varepsilon \right]\}dV \end{array}$$

### 2.2. Materials and Implementation

Several material parameters are required for the material models of the various materials included in the molding assembly. For the metallic components of the simulation, material properties were sourced from the material suppliers (SKF Sweden AB, Gothenburg, Sweden and Monitec Verkstads AB, Gothenburg, Sweden) [35,36]. These properties include thermal conductivity, density, Young’s Modulus, Poisson’s Ratio, coefficient of linear thermal expansion, and specific heat capacity. The metallic components are simulated using simple elastic material models as no plastic deformation was observed in either the tests or the simulations and the stresses calculated are not near the elastic limit of the materials.

Care was taken to calculate appropriate material properties for the silicone rubber material model used in the analysis, which can be found in Table 1. The values for density, coefficient of thermal expansion, thermal conductivity, specific heat capacity, interface conductance with the adjacent metallic surfaces, and the mechanical properties are discussed in detail in the following paragraphs.

Density is a function of temperature, but, in this analysis, the density measured at room temperature is used as a constant and the variation in volume is instead dealt with using the thermal expansion parameters. The room temperature density of the silicone rubber is calculated directly from the mass and volume measurements [29]. The calculated density is within the range of the density provided by the supplier (1.04–1.14 g/cm^3^) [37].

The coefficient of thermal expansion of the rubber was found to have a significant effect on the overall analysis results and therefore needed to be measured accurately. A thermomechanical analyzer (TMA) was used to measure the dimension change of silicone rubber upon heating [29]. For the measurement, the test was performed on a Q400 TA machine (TA Instruments, New Castle, DE, USA) in standard mode in the temperature range of 30–200 °C. A constant force of 0.05 N was applied during measurement and the heating rate was 5 °C/min. Additionally, the coefficient of thermal expansion measured lies within the expected range of silicone rubbers (250–300 × 10^−6^/K) [38].

The thermal conductivity was derived from the TRP characterization test results. Equation (3) is derived from the steady state heat flow experiment by [39]. Here, κ is the thermal conductivity. $T_{c}$ is the temperature in the rubber at 2 mm outside of the interior stainless steel rubber interface taken after 60 min, $T_{s}$ is the temperature in the rubber at 4 mm outside of the interior stainless steel rubber interface taken after 60 min, and $T_{o}$ is the temperature in the rubber at 4 mm outside of the interior stainless steel rubber interface taken after 30 min. The time span of 30–60 min is used to derive the thermal conductivity because, in this range, the heat conduction is highest, which leads to a more precise determination. The computed thermal conductance fits well with the expected result [29].

(3) $$\kappa =\ln\left(\frac{T_{c}-T_{s}}{T_{o}-T_{s}}\right)/\left(rt\right)$$

The specific heat capacity of silicone rubber is between 1050 and 1300 J/kgK [38]. Changes in the specific heat capacity do not have much effect on the response of the simulated results and therefore an approximate average value was chosen. Initial simulations used both minimum and maximum values available in literature and there were no discernable differences in either heat conduction or change in surface pressure. The specific heat capacity of the silicone rubber is modeled independent of pressure, as changes are insignificant for the analysis.

The heat transfer at the interface is complex and depends on many physical properties [40]. Thermal interfacial contact conductance properties [41] are required to simulate contact between the different internal material surfaces in the processing mold simulation. Tabular thermal conductance is used, with only pressure-dependent data. The variation of thermal contact conductance with the amount of pressure at the contact surface is directly included in the contact property. Two contact surfaces are defined for the contact between the aluminum and the rubber and the contact between the stainless steel and the rubber. Table 2 provides the detailed gap conductance and contact conductance used for the two surface pairs. The first contact pair consists of the inner surface of the aluminum mold and the outer surface of the rubber and the second contact pair comprises the contact between the inner surface of the rubber and the outer surface of the stainless steel sphere. A surface-to-surface contact interaction is defined, and no interference is allowed for each contact pair. Additionally, convection has been accounted for assuming the gaps are filled with natural atmospheric gases. Here, the flow of heat is approximated as if between two large planar surfaces, because the radius of curvature of the mold is very large when compared with the spacing between contacts [41]. The surface roughness of the aluminum mold at the rubber interface was 0.8 μm and the surface roughness of the stainless steel sphere was approximately 0.01 μm. Because of the relatively low surface roughness of the stainless steel sphere, there is a possibility that the selected literature values give an underestimate of the interface conductance. However, in this study, the interface conductance was not directly measured. Based on literature, values in the range between 0.1 and 1 MPa [42] and a logarithmic relationship between contact conductance and pressure are applied for the simulation [43,44].

Mechanical properties for the silicone rubber material model were calculated based on the required response for the trapped rubber processing scenario. A Mooney–Rivlin rubber material model was chosen to characterize the hyperelastic response of the rubber material. The elastic modulus and the Poisson’s ratio are initialized using rubber supplier material data [37]. Using the approximation for a neo-Hookean material reduces the required constants to only two for each increment of temperature, Equations (4) and (5). One of the limitations of using the Mooney–Rivlin [30,31] material model available in Abaqus/Standard is that it can only be used with large deformation theory [34]. Large deformation theory is not required to model trapped rubber processing with cavity shaped silicone rubber components. Comparing large deformation theory and small deformation theory results when using a perfectly elastic material and there is less than 2% change in the surface pressure; therefore, the modification is considered stable but may add unnecessary computational time.

(4) $$\lambda =2\cdot D_{1}$$

(5) $$\mu =\;2\cdot C_{1}$$

An initial estimate for the Mooney–Rivlin coefficients can be determined by solving for the Lamé constants at room temperature using Equations (4) and (5). At room temperature, these coefficients can be calculated based on the initial elasticity with small deformation. Based on this assumption, Equations (6) and (7) can then be formulated. Equations (6) and (7) can be solved simultaneously to calculate the neo-Hookean, Mooney–Rivlin coefficients (*C*_1_ and *D*_1_) seen in Table 1.

(6) $$\nu =\frac{3-2\;C_{1}D_{1}}{6+2\;C_{1}D_{1}}$$

(7) $$E\;=\;\frac{2\;C_{1}\left(6\;C_{1}+6\;D_{1}\right)}{2\;C_{1}\;+\;2\;D_{1}}$$

These Mooney–Rivlin coefficients can be defined at an array of discrete temperatures for the material; however, these materials have reasonably stable thermal conductivities at 25 and 400 °C [45], and the coefficients are therefore kept constant throughout the temperature range studied.

### 2.3. Evaluation

The model is constructed to effectively represent the test apparatus [29] including the full mold for simulation. Due to symmetry, only one eighth of the temperature change test is simulated. Because of the high thermal conductivity of the aluminum cavity mold, the interior temperature at the rubber surface can be assumed to be uniform. The simulations confirm this and show less than 0.0001 °C of a difference in temperature on the inner cavity surface of the aluminum mold during the full simulation. In order to compare directly with the readings from the five thermocouples that are distributed in the rubber, nodes are placed at these through-thickness locations. Nodal temperatures can then be extracted directly from the model and compared with the experimental results. From the simulation, the Abaqus variable CPRESS (normal force from contact pressure) is evaluated to compare with the experimental results [34].

### 2.4. Experimentation

The model is calibrated based on previously collected experimental data. A method for experimentally characterizing prospective rubber materials was developed [29]. The experiments were designed to characterize the dynamic in situ change in temperature, the dynamic change in volume, and the resulting real-time change in surface pressure. The material characterization was specifically designed to minimize the number and difficulty of experimental tests while capturing the rubber behavior for the TRP scenario. Both in situ temperature of the rubber and pressure at the internal surface of the rubber and the outer aluminum clamshell mold were collected over a thermal transient ranging from 5 to 22.3 °C [29].

## 3. Results and Discussion

The simulation results compare well with the experimental tests when the gap is calibrated via simulation optimization. The specific gap or gaps between the hard surfaces in contact with the rubber dominate the pressure response.

### 3.1. Modeling

**Figure 4:**
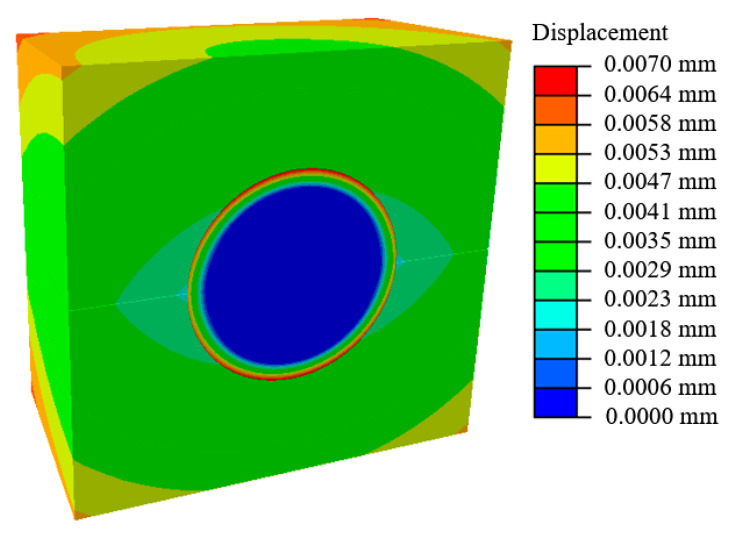
Displacement field map of the assembly cross section at temperature equilibrium (22.3 °C).

A model has been constructed to simulate the experiments. Both the in-situ temperature change and pressure change at the internal surfaces are evaluated from the simulation using the method described above. A cross-sectional view of half the assembly can be seen in Figure 4. The stainless steel sphere undergoes very little change in dimensions, while the aluminum and rubber sections expand more due to the change in temperature. Figure 4 visually demonstrates the internal tooling assembly motion during testing. In this figure, the initial gap between the external surface of the rubber and the internal surface of the aluminum clam shell is 0.007 mm and the rubber has expanded to fill this entire gap. Additionally, the seam between the clam shell molds is visible because the pressure is pushing the mold halves apart causing them to separate slightly. A smaller gap of 0.007 is used here because the higher resulting pressures accentuate the gap for visibility. This separation movement is reduced near the bolted connections and one of these locations can be seen on the side.

#### 3.1.1. Temperature Change

**Figure 5:**
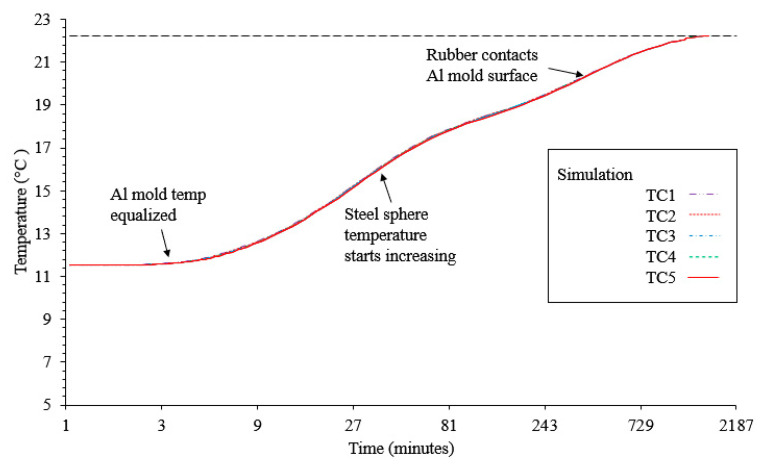
Through-thickness temperature distribution over time.

A temperature boundary condition was prescribed on all exterior assembly surfaces. A linear ramp in temperature from 11.2 to 22.3 °C over 200 s is used to correspond with the temperature change observed in testing [29]. A small thermal delta with an initial temperature below room temperature was used to ensure the pressure sensors would not reach capacity. Using this simplification, convection was not modeled on the exterior surface. This may cause a more rapid temperature increase in the simulation compared with the experimental results. However, this difference will not affect the final pressure observed. It can be observed that the aluminum mold distributes the heat well from this simulation. The thermal results from the simulation through the thickness of the rubber can be seen in Figure 5. Based on the simulation shortly before the aluminum mold reaches a stable temperature, the rubber starts heating. The rise in temperature over time is slightly reduced as the internal stainless steel sphere starts heating. There is not much difference in the temperature over time between the different assembly components once the rubber comes in contact with the aluminum.

#### 3.1.2. Pressure Change

**Figure 6:**
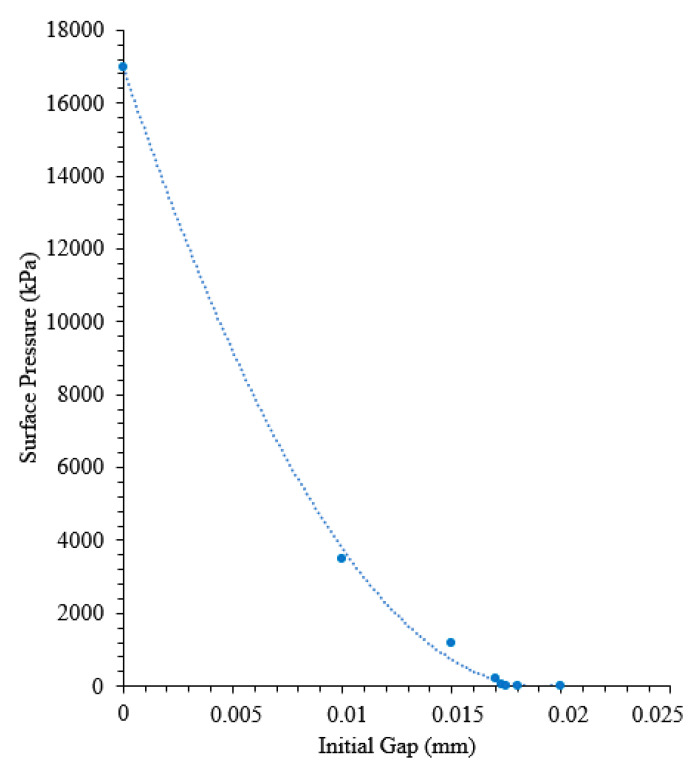
Maximum surface pressure (kPa) as a function of the initial gap between the Al mold and rubber.

In order to initialize the gap between the aluminum mold and the rubber, gap correlation simulations were conducted. The approximate average gap was found to be 0.017 mm for the corresponding experimental tests. Because of the construction method, it was assumed that there was no gap between the stainless steel and rubber surfaces. The results for the gap correlation can be seen in Figure 6. The gap shown here is the distance from a point on the outer surface of the rubber sphere to the closest point on the inner surface of the aluminum mold if the rubber was exactly concentric with the mold cavity when being suspended. This is an idealized case, and gravity is omitted for simplicity. As the gap between the surfaces decreases, the maximum surface pressure at thermal equilibrium increases sharply. Thus, the maximum surface pressure is highly sensitive to the dimensions of the mold assembly components. This highly nonlinear effect is one of the reasons why it is difficult to optimize TRP using a trial and error approach.

**Figure 7:**
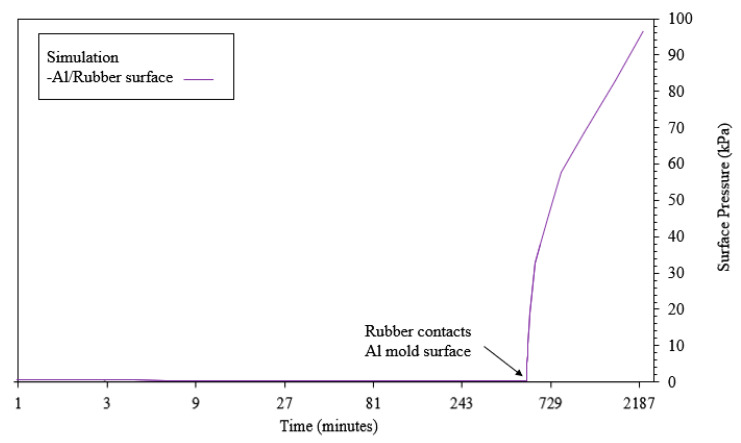
Rubber Surface Pressure (kPa) over time.

The computed surface pressure can be seen in Figure 7. As mentioned above, a linear ramp in temperature from 11.2 to 22.3 °C on the mold exterior is used. Shortly after the rubber contacts the mold cavity surface, the pressures start to increase rapidly. As the pressure increases above 60 kPa, some small elastic deformation can be observed near the bolted connections at the four corners of the cubic clamshell mold.

### 3.2. Validation

**Figure 8:**
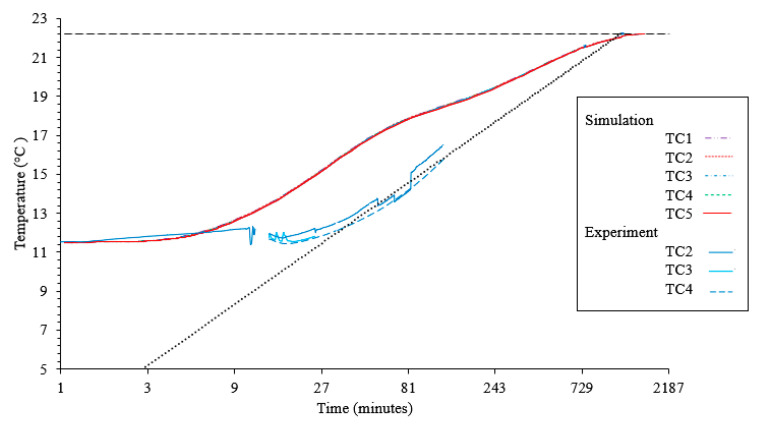
Comparison of simulated thermal change with experimental data.

**Figure 9:**
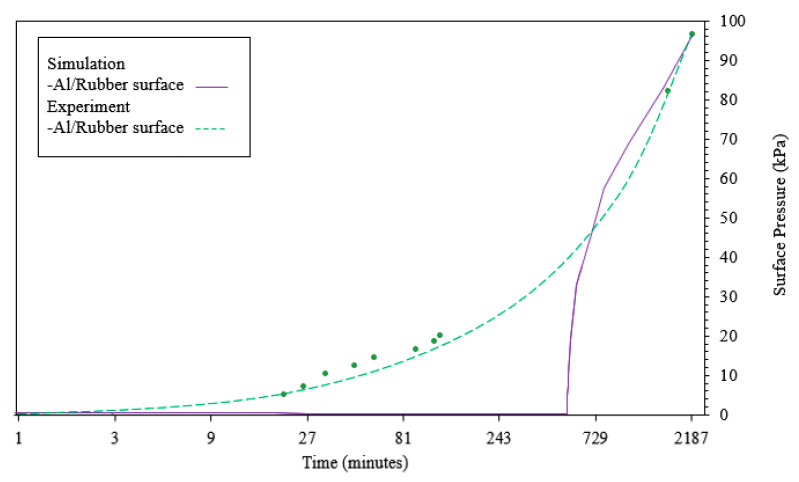
Comparison of simulated pressure change with experimental data.

The simulation results are compared with those of the experiments regarding internal rubber temperature and external surface pressure of the rubber. The internal temperature comparison can be seen in Figure 8. There is a slightly flatter curve observed in the computed results compared with the experimental. The more rapid increase in temperature observed in the simulation could be due to the simplifications regarding the exterior boundary condition. Additionally, there is very little temperature difference between the five different through thickness locations investigated. The raw data seems to indicate that more difference should be expected between the individual temperature interrogation points. However, when the expected thermocouple deviation is included [29], the range of temperatures in simulation compares well. The surface pressure on the external surface of the rubber comparison can be seen in Figure 9. Shortly after the rubber contacts the mold cavity, the pressures agree well.

### 3.3. Sensitivity Analysis

The sensitivity of the unknown parameters has been studied to better understand how to design TRP processes and can be seen in Table 3. An inverse parameter identification was performed for the initial gap as discussed in Section 3.1.2. This parameter was found to have the most significant effect on the resulting surface pressure. The interface thermal conductance was calculated from values found in literature as discussed in Section 2.2. While the range studied is somewhat unrealistic, it can be observed from the findings that these parameters are important to matching the dynamic phases of the thermal transient. The specific heat capacity used was found in literature, as discussed in Section 2.2. The range of values investigated did not yield any significant changes in the output of the simulation.

### 3.4. TRP Design

**Figure 10:**
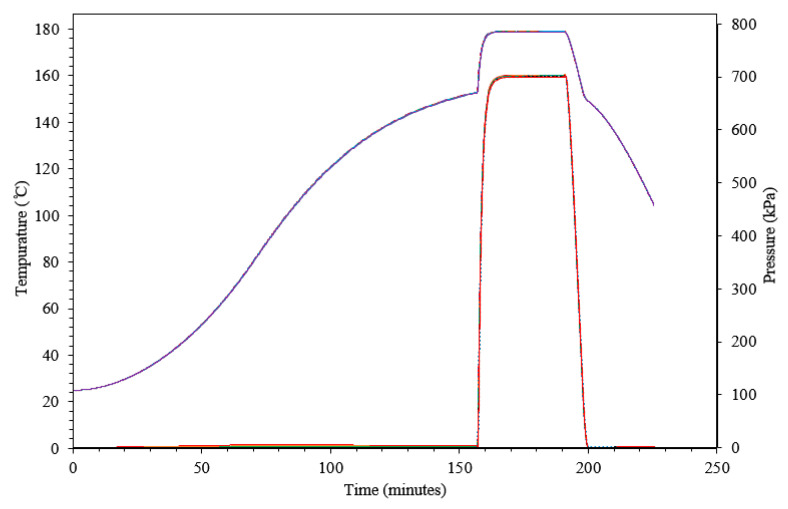
Internal rubber temperature (**violet**) and surface pressure (**red**) over the processing cycle time.

The model and its parameters described above were then used to simulate part processing in advanced trapped rubber molding. A similar TRP assembly can be used to process thermoplastic composite tape or thermoset pre-impregnated fibers (prepreg). An example is simulated for use with a prepreg that has an optimal cure temp of 180 °C cure pressure of 700 kPa (7 bar) and a ramp rate of 2 °C min. Here, the gap has been determined via simulation optimization to be 0.19 mm for the previously described experimental assembly. Based on this, the cure cycle can be simulated as seen in Figure 10. The full analysis took 422 s on a local computer with an Intel^TM^ i7-7600U CPU running at 2.80 GHz. The simulation boundary conditions have prescribed heat-up, dwell and cool-down steps. The resulting predicted temperature of the rubber can be seen in Figure 10 as the violet curve and the resulting predicted surface pressure is seen as the red curve. The resulting processing cycle fits with many common autoclave resins [5]. It can also be seen that a post cure without pressure would be possible at 170 or 175 °C. This example does not simulate the composite prepreg material. However, it is expected that current simulation techniques for composite processing [10] can be combined with the TRP simulation to yield a good approximation of the in-situ resin temperature and pressure during the cycling.

## 4. Conclusions

In this paper, a straightforward extraction of parameters for a finite element material model for TRP materials has been established. An integrated computational material design framework is proposed that allows for the detailed and systematic experimental characterization of parameters.

There exists a broad industrial need for alternatives to autoclave high-performance composite processing that allows for faster throughput without relinquishing performance. Currently, manufacturing methods for continuous fiber composites for high performance is dominated by intuitive manufacturing techniques that can be improved experimentally via an intensive trial and error process. In order to move beyond this trial and error regime, robust simulation methods of processing must be formulated.

TRP is one of these less intuitive processing techniques. The development of a robust TRP method has the potential to lead to more efficient and sustainable reinforced polymer composite manufacturing considering the reduced energy required for manufacturing and the reduced labor by reducing the trial and error process and reducing assembly time. One of the main advantages of TRP is the flexibility to use complex molds that include ribs and stringers. This all-in-one technique can vastly reduce the typical assembly time required to bolt or adhere these individual components together. There are three key achievements in the current study.

(1) A computational model has been developed based on the previous experimental characterization [29] of TRP. An efficient fully coupled thermomechanical simulation has been formulated from a combination of tested and calculated material and manufacturing assembly parameters. The use of reduced integration elements in both metal and rubber sections reduced the total simulation runtime by approximately 30%, with minimal influence on surface pressures (<1%).
(2) A sensitivity analysis has been conducted on the model to established significant parameters in TRP. The most significant variable in TRP design, based on this investigation, is the designed gap, or spacing between the assembly components. The maximum pressures obtained are highly dependent on the gap. Too small a gap between rubber and mold resulted in large pressures and elastic deformations of the mold; such distortions are often reported as the failure mode of TRP developed through trial and error. A computational model, such as the one presented in this paper, can be used to optimize the gap via inverse parameter identification. Additionally, based on this finding it is recommended that a high-precision mold is used for curing the silicone rubber and that shrinkage is accounted for during the initial curing step.
  The thermal conductivity is also a significant parameter in the simulation. In this paper, thermal conductivity is back-calculated based on previous experimental data. This method relies on the assumption that the material is homogeneous. For nanocomposite TRP materials, it may be important to measure thermal conductivity more precisely, using techniques such as laser flash analysis (LFA).
(3) A TRP design process has been simulated for a well-established autoclave cure continuous fiber composites prepreg. Using the fully coupled computational model developed in this paper, the full processing simulation was run in less than 10 min on a local computer.

## Figures and Tables

**Table 1:** Silicone rubber material (Mooney–Rivlin hyperelastic).

Property	Value	Units
Mooney–Rivlin (C1)	2.50 × 10^8^	Pa
Mooney–Rivlin (D1)	8.05 × 10^−11^	Pa
Density	1.049	g/cm^3^
Conductivity	1.9	W/mK
Specific Heat Capacity	1050	J/kgK
Coefficient of Thermal Expansion	286 × 10^−6^	/K

**Table 2:** Thermophysical contact properties used in the numerical simulation.

	Stainless Steel/Rubber Interface		Aluminum/Rubber Interface	
	Conductance (W/K)	Clearance (m)	Conductance (W/K)	Clearance (m)
Gap Conductance	1.05	0	4.07	0
	0.9	0.0025	0.9	0.0025
	0.038	0.005	0.038	0.005
	0	0.01	0	0.01
	Conductance (W/K)	Pressure (Pa)	Conductance (W/K)	Pressure (Pa)
Contact Conductance	1.05	0	4.07	0
	1.37	100	5.29	100
	1.78	700	6.88	700
	32.4	1.00 × 10^6^	126.25	1.00 × 10^6^

**Table 3:** Simulation sensitivity to unknown parameters.

Parameter	Minimum	Maximum	Response
Initial gap	0.00 mm	0.02 mm	Changes in final pressure from 17,000 to 0 kPa (Figure 6).
Silicone rubber thermal conductivity	0.2 W/mK [38]	2.55 W/mK [38]	No change in final pressure, the difference in temperature from the inner surface to the outer surface of the rubber changes from 1.0 °C at the minimum to 0.05 °C at the maximum value.
Interface thermal conductance	0.9 W/K	250 W/K	No change in final pressure. Using the maximum value, the rubber temperature increase starts 80% earlier and exponent is increased by a power of one compared to the minimum value.
Specific heat capacity	1050 J/k [38]	1300 J/k [38]	No change in final pressure, less than 0.01% change in the temperature over time.
